# Antibiotic Prescribing Patterns at COVID-19 Dedicated Wards in Bangladesh: Findings from a Single Center Study

**DOI:** 10.1016/j.infpip.2021.100134

**Published:** 2021-02-27

**Authors:** Md. Maruf Ahmed Molla, Mahmuda Yeasmin, Md. Khairul Islam, Md. Mohiuddin Sharif, Md. Robed Amin, Tasnim Nafisa, Asish Kumar Ghosh, Monira Parveen, Md. Masum Hossain Arif, Junaid Abdullah Jamiul Alam, Syed Jafar Raza Rizvi, K.M. Saif-Ur-Rahman, Arifa Akram, A.K.M. Shamsuzzaman

**Affiliations:** a National Institute of Laboratory Medicine and Referral Center, Dhaka, Bangladesh; b Dhaka Medical College and Hospital, Dhaka, Bangladesh; c Dhaka Dental College, Dhaka, Bangladesh; d Evercare Hospital, Dhaka, Bangladesh; e Johns Hopkins University, Center for Communication Programs, Dhaka, Bangladesh; f Health Systems and Population Studies Division, icddr,b, Dhaka, Bangladesh

**Keywords:** COVID-19, Antimicrobial resistance, Bangladesh, SARS-CoV-2, Point prevalence survey

## Abstract

**Background:**

As evidence is mounting regarding irrational and often unnecessary use of antibiotics during the COVID-19 pandemic a cross-sectional Point Prevalence Survey (PPS) (in accordance with WHO guideline) was conducted across COVID-19 dedicated wards in Dhaka Medical College and Hospital (DMCH).

**Methodology:**

Antibiotic usage data were collected from 193 patients at different COVID-19 dedicated wards at DMCH on 11 June 2020. Comparisons in antibiotic usage were made between different groups using Pearson chi-square and Fisher's exact test.

**Result:**

Findings reveal all surveyed patients (100%) were receiving at least one antibiotic with 133 patients (68.91%) receiving multiple antibiotics. Overall, patients presenting with the severe disease received more antibiotics. Third-generation cephalosporins (i.e. ceftriaxone) (53.8%), meropenem (40.9%), moxifloxacin (29.5%), and doxycycline (25.4%) were the four most prescribed antibiotics among surveyed patients. Diabetes mellitus (DM) was independently associated with multiple antibiotic prescribing. Abnormal C-reactive protein (CRP) and serum d-dimer were linked with higher odds of multiple antibiotic prescribing among study patients.

**Conclusion:**

Prevalence of multiple antibiotic prescriptions was high among severely ill patients and those with abnormal CRP and d-dimer levels. Data regarding the quality of antibiotic prescribing were lacking.

## Introduction

The COVID-19 pandemic has led to an unprecedented crisis in every aspect ofhealthcare systems across the world. Most infected people presenting with mild to moderate symptoms (such as cough, fever, and lung infiltrates) display symptoms resembling bacterial pneumonia. Only 20% of affected people get severe infection, and 6% of people who become critically ill require ICU support [1]. Despite the viral origin of COVID-19 and lack of evidence of bacterial superinfection in a huge number of cases, physicians are often compelled to prescribe a plethora of antimicrobials due to lack of specific antiviral treatment and vaccine against SARS-CoV-2, difficulties in differentiating between bacterial pneumonia and COVID-19, and uncertainty regarding secondary bacterial infection [2,3]. Different studies revealed that 70% of hospitalized patients receive one or more antibiotics, this rises to 100% in the ICU setting [2,3]. Excessive prescribing and overuse of antibiotics is notable during this pandemic; in the long run this may complicatemanagement of COVID-19 patients and the existing battle against antimicrobial resistance (AMR) [4,5]. Thus far, data on hospital antibiotic consumption and prescribing patterns during the COVID-19 pandemic are sparse, especially in countries without a well-functioning antimicrobial stewardship program. Point prevalence surveys (PPS) on antibiotic usage among hospitalized patients reflect the actual scenario of antibiotic prescribing and will aid in strategic planning of antibiotic stewardship programs. This is particularly true in countries like Bangladesh, where there is widespread ignorance among the general population and health care providers regarding antibiotic overuse, resistance and how it may impact the future of healthcare. This single-center cross-sectional study conducted at Dhaka Medical College and Hospital (DMCH), will fill a knowledge gap and aid in the proper planning of COVID-19 clinical management.

## Materials and methods

### Study design and subjects

This single-center cross-sectional PPS study was conducted at COVID-19 dedicated wards (excluding critical care units) at DMCH on June 11 and the surverycomprised of patients admitted between May 21 and June 10, 2020. DMCH, a 2,300 bed tertiary level teaching hospital and the largest government run hospital in Bangladesh. Among the patient beds, 883 are reserved for COVID-19 patients and current occupancy aon COVID-19 wards stands at 50%, with an average between 20-50% since the inauguration of COVID-19 dedicated wards in April 2020. For this study, data were collected from a total of 227 COVID-19 patients. After excluding entries with missing data, 193 patients were included in the final analysis.

Adult patients (≥18 years) with a confirmed SARS-CoV-2 PCR positive result were considered for this study. Suspected COVID-19 patients or patients awaiting their rt-PCR results were excluded from the study.

### Control group selection

During the peak transmission period of the COVID-19 pandemic, all patients at DMCH received a broad-spectrum antibiotic upon admission on a routine basis. Hence, no control group could be established comprising of patients who did not receive any antibiotic during their hospital stay.

### Clinical definition of COVID-19 patients

On admission, patients were categorized into mild/moderate/severe or critical groups based on criteria set by World Health Organization (WHO). For this study, patients on COVID-19 dedicated wards at DMCH were identified as suffering from either moderate or severe disease.

### Data regarding antibiotics prescription

Since this study specifically deals with antibiotics, antimicrobials including anti-viral, anti-parasitic, and anti-fungal agents were excluded from the final analysis. Topical applications were excluded from the antibiotic list. Patients were divided into two groups – patients with no or a single antibiotic and patients with multiple antibiotics on their treatment sheets on the data collection date.

### Co-morbidities and biochemical markers

Apart from demographic information, co-morbidities including hypertension (HTN), diabetes mellitus (DM), ischemic heart disease (IHD), asthma, chronic obstructive pulmonary disease (COPD), chronic kidney disease (CKD), and pre-existing malignancy history were recorded. Patients were categorized into two groups – patients with no or a single comorbidity and patients with two or more comorbidities.

Biochemical marker values, specifically ones associated with disease severity and antibiotic prescribing, such as C - reactive protein (CRP), d-dimer, and serum ferritin level were recorded from investigation files and were expressed as mean ± SD. Biochemical markers were loosely divided into two categories based on findings – normal and abnormal. Following values were considered as normal for aforementioned biochemical markers – d-dimer (≤0.5 μgm/ml considered as negative screening), and serum ferritin (for men 24–336 μgm/l; for female 11–307 μgm/l). Values outside of the normal range were grouped as abnormal biochemical findings.

Regarding CRP, internationally recognized protocol for CRP guided antibiotic therapy constitutes of following parameters: <20 mg/L for withholding treatment, using discretion between 20-80 mg/L, and immediate initiation of antibiotic if surpasses >100 mg/L. For this study, considering the immediate initiation of antibiotic on all patients and empiric nature of COVID-19 treatment, CRP was loosely divided into two categories - <20 mg/L as normal and not requiring antibiotic therapy, and ≥20 mg/L as abnormal and requiring antibiotic therapy.

### Data management and analysis

For this study, statistical analysis was done using Statistical Package for the Social Sciences (SPSS) software version 25. Continuous variables were expressed as mean ± SD and categorical variables were expressed in numbers (n) and percentage (%). Fisher's exact and Pearson's chi-square tests were used to test the association between variables. For all analyses, statistical significance was set at *P*<0.05.

### Ethical consideration

For this study, researchers collected all the data from patient history sheets and anonymous data were sent to the core team for statistical analysis. No patient was interviewed during the study and hence informed written consent was waived. This study was approved by the ethical review board at the National Institute of Laboratory Medicine and Referral Center (NILMRC), Dhaka, Bangladesh (NILMRC/IRB/2020/05).

## Results

### Demographic characteristics and disease severity categorization

For this survey study, 193 patients were enrolled, of whom 134 (69.4%) were male and 59 (30.6%) were female. Patient age ranged from 18 to 95 with the mean age of participants being 50.44±14.08 years. Patients' clinical condition was either categorized as moderate or severe upon admission (*P*=0.128). No statistical association could be observed between gender and disease severity (*P*=0.948) (Table- 1).

**Table-1:** Age and sex distribution and clinical categorization of patients

Age groups	Frequency (n=193)	Moderate (n=89)	Severe (n=104)	p-value
18–25	11 (5.70%)	7 (7.9%)	4 (3.9%)	
26–35	22 (11.40%)	12 (13.5%)	10 (9.6%)	
36–45	39 (20.21%)	16 (18%)	23 (22.1%)	0.146
46–55	51 (26.42%)	27 (30.3%)	24 (23.1%)	
56–65	48 (24.87%)	22 (24.7%)	26 (25%)	
66+	22 (11.40%)	5 (5.6%)	17 (16.4%)	
Male	134 (69.43%)	62 (69.66%)	72 (69.23%)	0.948
Female	59 (30.57%)	27 (30.34%)	32 (30.77%)	

### Antibiotic prescribing pattern

All study patients (100%) received one or more antibiotics on the survey date. In total, 193 study participants received a total of 389 antibiotics, with each patient receiving 2.01 antibiotics on average, from the time of hospital admission to the survey date. Among them, 60 patients (31.08%) received a single antibiotic agent whereas the remaining 133 patients (68.91%) received two or more antibiotics on the survey date. Ceftriaxone, a beta-lactamase stable broad-spectrum antibiotic, was found to be the highest prescribed drug with 104 patients (53.88%) out of 193 total participants receiving the drug according to their treatment record (Table- 2). Secondly, Meropenem, another broad-spectrum injectable antibiotic, was prescribed in 79 (40.9%) surveyed patients (Table- 2). Doxycycline, an oral antibiotic, was prescribed in 49 patients (25.4%) (Table- 2). All prescribed antibiotics recorded during this survey were broad-spectrum in nature.

**Table-2:** Association between antibiotics prescribed and disease severity

Antimicrobial	Moderate (n=89)	Severe (n=104)	Total (n=193)	p-value
**Ceftriaxone**	**55 (61.8%)**	**49 (47.11%)**	**104 (53.88%)**	**0.028**
Meropenem	23 (25.8%)	56 (53.9%)	**79 (40.9%)**	**<0.001**
Levofloxacin	13 (14.6%)	14 (13.5%)	27 (14%)	0.819
Moxifloxacin	22 (24.7%)	35 (33.7%)	57 (29.5%)	0.177
**Doxycycline**	**16 (18%)**	**33 (31.7%)**	**49 (25.4%)**	**0.029**
Azithromycin	22 (24.7%)	23 (22.1%)	45 (23.3%)	0.672
Amoxicillin	6 (6.7%)	14 (13.5%)	20 (10.4%)	0.128

### Comorbidity, inflammatory markers, and disease severity

Results revealed a statistically significant association between DM and the number of antibiotics received per patient (*P*=0.007). No statistical association could be found between multiple antibiotic prescribing among patients and other comorbidities (Table- 3). Besides, patients with multiple comorbidities were more likely to present with severe disease (*P*=0.005) but no statistical significance could be found between multiple comorbidities and increased antibiotic prescription (*P*=0.056) (Table- 4). Additionally, there was a significant correlation between disease severity and the number of antimicrobials received per patient (*P*=<0.00001).

**Table-3:** Comorbidity and antimicrobial prescribing

Comorbidity	Antibiotic (0–1) Yes/No (n=60)	Antibiotic (≥2) Yes/No (n=133)	p-value
Hypertension	25/35	70/63	0.160
**DM**	**16/44**	**63/70**	**0.007**
IHD	12/48	38/95	0.208
Asthma	13/47	20/113	0.258
COPD	5/55	14/119	0.636
CKD	3/57	11/122	0.417
Malignancy	0/60	2/131	0.474^a^

a Analyzed using 1-tail Fisher's exact test.

**Table-4:** Association between total number of comorbidities and disease severity, antibiotic prescription

	Total comorbidity (0–1)	Total comorbidity (≥2)	p-value
Antibiotic (0–1) (n=60)	36	24	
Antibiotic (≥2) (n=133)	60	73	0.056
Moderate disease (n=89)	54	35	
Severe disease (n=104)	42	62	**0.005**

Statistically significant association was found between disease severity and CRP (*P*=0.041), serum ferritin (*P*=0.022), and d-dimer level (*P*=0.006) (Table- 5). Among the common inflammatory markers, only CRP (*P*=0.005) and d-dimer (*P*=0.002) showed a statistically significant association with multiple antibiotic prescriptions among study patients (Table- 5).

**Table-5:** Association between biochemical markers, disease severity and antibiotic prescribing pattern

Biochemical marker	Mean±SD value	Antibiotic number (0–1) Normal/Abnormal	Antibiotic number (≥2) Normal/Abnormal	p-value	Moderate disease Normal/abnormal	Severe disease Normal/abnormal	p-value
**CRP**	**(23.8 ± 27.2) mg/l**	**28/32**	**35/98**	**0.005**	**55**/**34**	**49**/**55**	**0.041**
**d-dimer**	**(1.3 ± 1.5) μgm/ml**	**29**/**31**	**34**/**99**	**0.002**	**38**/**51**	**25**/**79**	**0.006**
Serum ferritin	(630.8 ± 664.8) μgm/l	17/43	31/102	0.455	29**/**60	19**/**85	0.022

## Discussion

Current guideline from WHO indicates that no antibiotic or antifungal drug should be prescribed in mild or moderate cases unless there are pre-existing symptoms of bacterial or fungal co-infection [6]. Furthermore, regarding empirical antimicrobial prescription in severe cases, patients' overall health condition, local epidemiology and the clinical judgment from the treating physician should be integrated to allow for judicial antimicrobial usage [6].

In Bangladesh, during the early months of the pandemic, there were reports of widespread antimicrobial consumption among COVID-19 positive and suspected patients-in most cases even without a prescription from a certified physician. In a previous study conducted among Bangladeshi COVID-19 patients isolating at home with mild or asymptomatic infection, 63% received one or more antimicrobial agents, including investigational drugs such as ivermectin, remdesivir, and favipiravir [7].

All patients admitted to DMCH during the peak transmission period routinely received broad-spectrum, either injectable or oral, antibiotics upon admission. Hence, it could not be ascertained whether the antibiotic usage was high or not, especially without a proper control group any argument in favor or against would be flawed. Nevertheless, a PPS conducted in different hospitals in Scotland and Singapore during April 2020 revealed at least one antimicrobial, which included antivirals and antifungals, was prescribed in 38.3% and 6.2% hospitalized SARS-CoV-2 positive patients (which included critical care units) respectively [8,9]. There is a well-functioning Scottish Antimicrobial Prescribing Group (SAPG), established in 2008, tasked with overseeing the pattern of antimicrobial usage in Scottish hospitals and implement antimicrobial stewardship programs in different hospitals [10]. Likewise, antibiotic prescribing in Singapore hospitals is highly regulated by antimicrobial stewardship units and often supplemented by relevant laboratory investigations [11]. In contrast, there is no such governing body in Bangladesh, both in government and private hospitals, and antimicrobial usage in hospitals across the country is largely empirical.

With a modest healthcare budget and huge patient turnover, it is tough for government-run hospitals to perform modern biochemical tests such as procalcitonin, serum ferritin, CRP, d-dimer, or to send samples for microbiological culture for each hospital admitted patient [12]. Hence, physicians often have to rely solely on their clinical experience and provide symptomatic management even before receiving laboratory confirmation of infection. This may justify the early initiation of antibiotic prescribing among study patients.

Among all listed comorbidities, DM was independently associated with increased antimicrobial usage. DM is a pre-infectious condition and previous studies suggest people admitted at hospitals with DM end up receiving more antibiotics than patients without DM, largely due to the presence of antibiotic resistance gene in the diabetic population [13]. This finding may have compelled physicians to prescribe multiple antibiotics to make sure the patient's condition does not worsen. In contrast, in the Scottish study COPD was identified to be positively associated and diabetes DM was negatively associated with antimicrobial usage. Regarding the Singapore study, patients with multiple comorbidities were more likely to receive antibiotics. In this study, no such association could be established between multiple comorbidities and increased antibiotic prescription.

Third-generation cephalosporins and meropenem were the two most prescribed drugs in the current study whereas co-amoxiclav, amoxicillin, and doxycycline were mostly prescribed in Scottish and Singaporean hospitals. The prevalence of multiple antibiotic prescribing during the survey period was higher compared to the Scottish and Singaporean studies. This can be explained by the fact that most patients admitted to DMCH have little financial means to purchase drugs from outside. As a result, they often rely on hospital-provided drugs and during the peak pandemic period, ceftriaxone and meropenem were supplied by the hospital free of cost.

Another valid explanation might be that all admitted patients in DMCH were either suffering from moderate or severe COVID-19 disease with most mild cases opting for home isolation, and injectable antibiotics are mostly prescribed in such cases to prevent the condition from deteriorating further [2]. The latest guideline of COVID-19 clinical management (version 7.00) published by the Ministry of Health and Family Welfare (MOHFW) advocates the use of meropenem in severely ill COVID-19 patients, which may have positively influenced meropenem prescribing on COVID-19 wards.

Inflammatory markers, such as CRP and d-dimer, performed after hospital admission, were found to be positively associated with both disease severity and multiple antibiotic prescribing among survey patients. This finding corroborates previous research findings where it was found that increased CRP and d-dimer is positively associated with poor patient outcomes [14].

There are several limitations to this PPS study. Antibiotic prescribing quality and appropriateness, an important component of PPS, could not be evaluated due to several factors including lack of valid data, immediate initiation of antibiotics on all patients irrespective of the presence of co-infection, lack of notes regarding antibiotic stop and review dates and absence of a prior national guideline on antibiotic use in hospitals. This was also a single-center study. For a comprehensive outlook on antibiotic usage in Bangladeshi hospitals, several other centers should have been included. Unfortunately, this was beyond the scope of this survey study, largely due to budgetary constraints. Future research into antimicrobial stewardship programs in Bangladeshi hospitals should consider these shortcomings and perform a comprehensive analysis of the overall antimicrobial usage situation in Bangladesh. Finally, the authors would like to propose several solutions to tackle this issue, as already proposed by others, such as reserving antibiotics for patients with severe presentations, obtaining microbiological evidence of co-infection beforehand, rapid re-evaluation of therapy after initiation, switching to oral drugs as soon as possible, opting for a short course of antibiotic (preferably not more than 5 days), stopping usage of prophylactic antibiotic in patients etc. [15].

## Author contributions

**Conceptualization**: MMAM, MY, TN, AKG, MMS, MRA; **Methodology**: MMAM, MKI, MRA, TN, AKG; **Data Collection**: MP, MMHA, JAJA, MMS, MRA, MKI; Writing: MMAM, MY; **Data Analysis**: MMAM, SJRR, KMSUR; **Review and Editing**: AA, AKMS, MRA, MKI, MMAM, MY; **Supervision**: MRA, MKI, AA, AKMS. All authors contributed significantly to this project and agreed on the final version of the manuscript before submission. All authors acknowledge that this is an original study and never submitted it for review previously.

## Confidentiality statement

The authors have nothing to disclose.

## Funding information

This research did not receive any specific grant from funding agencies in the public, commercial, or not-for-profit sectors.

## Data availability statement

If interested the readers can contact directly with the corresponding author for access to data and resources to replicate the findings discussed in this paper.
